# Multiscale dilated convolutional neural network for Atrial Fibrillation detection

**DOI:** 10.1371/journal.pone.0301691

**Published:** 2024-06-03

**Authors:** Lingnan Xia, Sirui He, Y-F Huang, Hua Ma

**Affiliations:** 1 Henan High-speed Railway Operation and Maintenance Engineering Research Center, Zhengzhou Railway Vocational and Technical College, Zhengzhou, China; 2 Department of Big Data Management and Application, Dalian Polytechnic University, Dalian, Liaoning, China; Menoufia University, EGYPT

## Abstract

Atrial Fibrillation (AF), a type of heart arrhythmia, becomes more common with aging and is associated with an increased risk of stroke and mortality. In light of the urgent need for effective automated AF monitoring, existing methods often fall short in balancing accuracy and computational efficiency. To address this issue, we introduce a framework based on Multi-Scale Dilated Convolution (AF-MSDC), aimed at achieving precise predictions with low cost and high efficiency. By integrating Multi-Scale Dilated Convolution (MSDC) modules, our model is capable of extracting features from electrocardiogram (ECG) datasets across various scales, thus achieving an optimal balance between precision and computational savings. We have developed three MSDC modules to construct the AF-MSDC framework and assessed its performance on renowned datasets, including the MIT-BIH Atrial Fibrillation Database and Physionet Challenge 2017. Empirical results unequivocally demonstrate that our technique surpasses existing state-of-the-art (SOTA) methods in the AF detection domain. Specifically, our model, with only a quarter of the parameters of a Residual Network (ResNet), achieved an impressive sensitivity of 99.45%, specificity of 99.64% (on the MIT-BIH AFDB dataset), and an $F_{1_{all}}$ score of 85.63% (on the Physionet Challenge 2017 AFDB dataset). This high efficiency makes our model particularly suitable for integration into wearable ECG devices powered by edge computing frameworks. Moreover, this innovative approach offers new possibilities for the early diagnosis of AF in clinical applications, potentially improving patient quality of life and reducing healthcare costs.

## Introduction

Atrial Fibrillation (AF), the most common cardiac arrhythmia in clinical practice, requires treatment intervention [1]. With the expanding aging population, the incidence of AF is also rising. However, due to an incomplete understanding of the pathophysiological mechanisms of AF, diagnosis has become challenging [2], particularly for patients with paroxysmal AF who require urgent attention. In the early stages, symptoms may be absent, and the arrhythmia may self-terminate [3]. It is estimated that traditional AF detection methods may fail to detect approximately 20% of AF cases [4–6]. In such cases, patients may not receive timely treatment, which can lead to adverse outcomes [7, 8]. Therefore, timely and accurate atrial fibrillation detection is particularly important.

Essentially, AF detection techniques can be categorized into three main types: those concentrating on atrial activity [9], those focusing on ventricular response [10], and those integrating both [11]. Atrial activity-based methods primarily focus on patterns like the absence of P-waves and the presence of f-waves but are sensitive to noise. On the other hand, ventricular response methods assess irregularities in RR intervals [12], with the QRS complex as a distinctive feature, making these techniques more robust [13]. By integrating atrial activity with ventricular response, the efficacy of AF detection can be amplified. However, the delineation of ECG waveforms, crucial for these analyses, remains vulnerable to noise interference. Typically, algorithms based on machine learning and deep learning extract features from both the atria and ventricles in ECG signals, commonly integrating the analysis of P waves and the irregularity of R-R intervals [14–16]. This approach falls under the category of comprehensive methods.

However, in traditional machine learning techniques, features representing cardiac arrhythmias are typically created through interaction with domain experts and through the review of relevant literature [17]. These features are then passed as input to shallow classifiers such as support vector machines (SVM) [16, 18, 19], and k-nearest neighbors [20]. These classifiers utilize these distinctive features to detect AF from ECG signals. For example, in the study by Henzel et al. [21], they input four statistical features of RR intervals into a generalized linear classifier for AF diagnosis. However, algorithms relying on manual feature extraction often overfit on training data, leading to poor performance when applied to unseen data [17]. This overfitting phenomenon may stem from manually selected features failing to fully capture the complex information in ECG signals, resulting in inadequate algorithm generalization [17]. Therefore, the application of traditional machine learning methods in the field of atrial fibrillation detection has been limited.

In recent years, deep learning (DL) technology has significantly surpassed traditional feature engineering and machine learning methods in multiple areas such as computer vision, natural language processing, and speech recognition, breaking through the limitations of conventional machine learning techniques [22]. Moreover, the application of deep learning in the detection of atrial fibrillation (AF) has showcased its tremendous potential for development, with an increasing adoption of deep learning technologies in AF detection [23, 24]. Specifically, convolutional neural networks (CNNs) have been extensively used as an important DL method in ECG signal analysis and classification [25–27]. Compared to traditional methods, CNNs can directly learn complex and representative features from data, thereby avoiding the need for manual feature engineering. For instance, Acharya et al. [28] presented an 11-layer CNN architecture with a four-neuron output layer for the classification of ECG signals [28]. Additionally, they constructed another 11-layer CNN model that effectively differentiates between shockable and non-shockable ventricular arrhythmias [29]. Rahhal et al. introduced an unsupervised DL approach for ECG classification and achieved encouraging results on publicly available databases including MIT-BIH and INCART arrhythmia databases [30]. Zubair et al. validated a CNN-based model on 44 ECG recordings from the MIT-BIH database, classifying ECG beats into five different categories [31]. These studies demonstrate the versatility and effectiveness of Convolutional Neural Networks (CNNs) in the analysis of electrocardiogram (ECG) signals and the detection of atrial fibrillation (AF). Compared to traditional methods of AF detection, deep learning (DL) approaches offer superior performance and robustness [32]. However, existing deep learning methods, despite their success in improving accuracy, typically face challenges related to high model complexity, significant computational resource consumption, and substantial storage requirements [33]. These issues often prevent achieving a good balance between accuracy and computational efficiency, limiting their application in resource-constrained environments. To address these challenges, this research introduces an efficient multi-scale dilated CNN framework aimed at exploring both lightweight and efficient deep learning algorithms. This approach, by combining multi-scale feature extraction with a lightweight network design, not only ensures the diagnostic accuracy of the model but also significantly enhances computational and storage efficiency, making it suitable for scenarios with limited computational resources.

**Fig 1:**
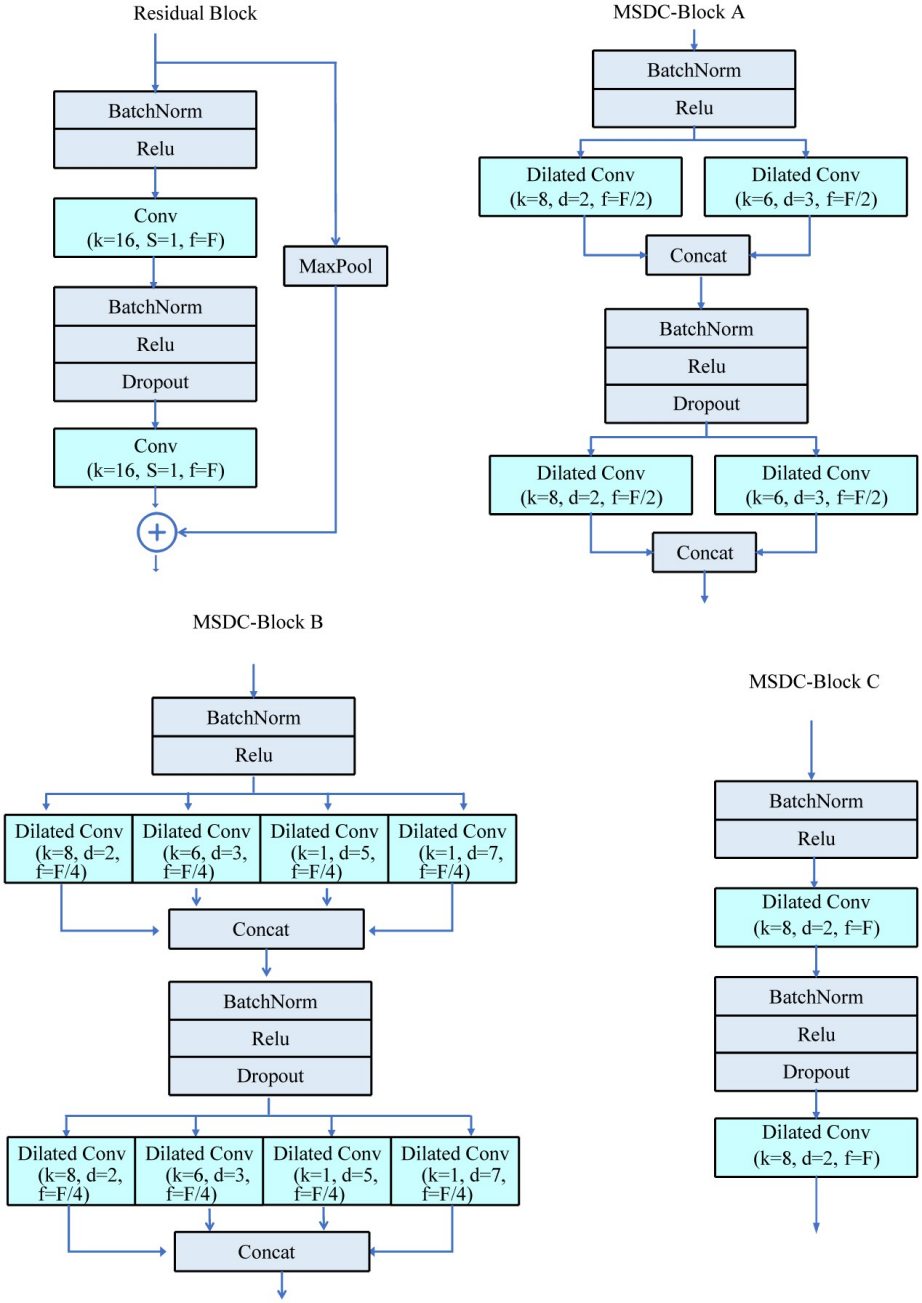
Comparison diagram of the three MSDC blocks and residual block structures. MSDC block C employs solely standard dilation, without the incorporation of multi-scale dilation convolution. Our proposed methodology introduces two distinct dilation convolution blocks, namely MSDC block A and MSDC block B. Comparative analysis of these convolution blocks, specifically focusing on their performance metrics, is detailed in Tables 3 and 5.

**Fig 2:**
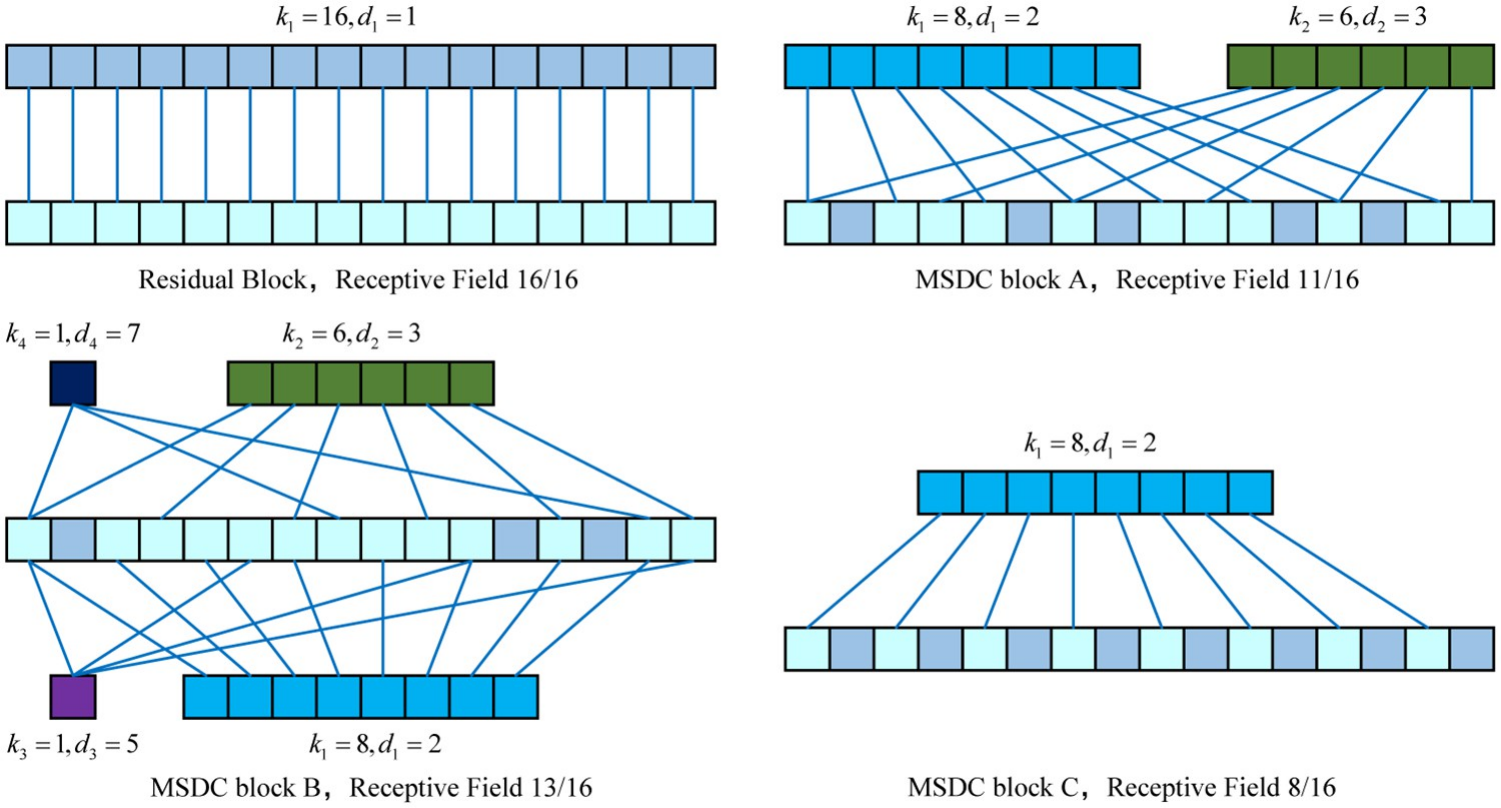
This figure illustrates how the receptive fields of three MSDC blocks and one residual block are calculated during a single convolution operation to generate the value of a single unit in the output feature map. As our objective is to predict atrial fibrillation from electrocardiogram signals, which are one-dimensional data consisting of 16 data points, the convolution kernels are utilized in a one-dimensional format.

The AF-MSDC (Atrial Fibrillation Detection using Multi-Scale Dilated Convolution) model proposed in this paper is an improvement on the 1D-ResNet network. Our key innovation lies in introducing Multi-Scale Dilated Convolutional blocks (MSDC blocks) to replace the traditional residual connection blocks in 1D-ResNet. MSDC blocks consist of multiple parallel dilated convolutional layers, which vary in the number of filters, kernel sizes, and dilation rates, thereby providing a rich multi-scale receptive field. This structural design not only maintains a receptive field size similar to that of traditional residual blocks (as shown in Fig 2) but also significantly reduces the model’s parameter count (as summarized in Table 1). We propose two multi-scale dilated convolution models, AF-MSDC A and AF-MSDC B (based on MSDC Block A and MSDC Block B from Fig 1), and have conducted comparative tests with a variety of state-of-the-art atrial fibrillation (AF) detection methods on two commonly used AF detection datasets, MIT-BIH AFDB and Physionet Challenge 2017, as well as ablation experiments with our proposed single-scale AF-MSDC C model (based on MSDC Block C from Fig 1). The AF-MSDC B model, while maintaining a receptive field comparable to the original residual block, has only a quarter of the parameter count of the 1D-ResNet model. It demonstrated a sensitivity of 99.45% and a specificity of 99.61% on the Physionet Challenge 2017 AFDB dataset, and achieved an F1 score of 84.83% on the MIT-BIH AFDB dataset, with performance similar to that of AF-MSDC A (which has a slightly higher parameter count than AF-MSDC B). These metrics surpass those of traditional 1D-ResNet models and other state-of-the-art models, indicating they can achieve exceptional AF detection performance with minimal storage space and computational power. Based on these advantages, the AF-MSDC models are promising candidates for embedding into wearable AF detection devices, relying solely on the limited resources of these devices for highly accurate detection, without the need for cloud-based computing, thus effectively avoiding risks such as data breaches.

**Table 1:** Parameter comparison between three MSDC blocks and residual block.

Blocks	(k, s, d, f)	Receptive field	Parameters
Residual block	(16, 1, 0, 32)	16	512
MSDC block A	(8, 1, 2, 16)(6, 1, 3, 16)	11	224
MSDC block B	(8, 1, 2, 8)(6, 1, 3, 8)(1, 1, 5, 8)(1, 1, 7, 8)	13	128
MSDC block C	(8, 1, 2, 32)	8	256

In summary, our contributions are summarized as the following:

1. **MSDC Block Innovation**: Introduced an efficient multi-scale dilation convolutional neural network (CNN) anchored by the novel MSDC block, offering a balance between performance and reduced parameter size.
2. **Benchmarked Superiority**: Validated our model on renowned datasets, achieving notable superiority over state-of-the-art models like ResNet in sensitivity, specificity, and $F_{1_{all}}$ score.
3. **Wearable Device Optimization**: Designed with wearables in mind, our model’s efficiency makes it ideal for real-time AF detection on edge computing-focused wearable ECG devices.

The subsequent sections of this paper are structured as follows: a comprehensive explanation of the proposed approach is presented in the Methodology section. The evaluation experiments are conducted, and their outcomes are discussed in the Experiments section. The paper concludes with a summary in the Conclusion section, along with a glimpse into potential future research directions.

## Related work

### Traditional Atrial Fibrillation detection algorithms

The importance of detecting atrial fibrillation (AF) is well-recognized, and the development of its detection algorithms is a topic of significant interest. Broadly, these algorithms fall into three categories:

1. Algorithms that analyze atrial activity [9].
2. Algorithms that focus on ventricular activity [10].
3. Hybrid algorithms that combine both atrial and ventricular activity [11].

Atrial activity in AF is primarily marked by the vanishing of P waves and the emergence of f waves. Detection based on this activity primarily relies on these features [34–38]. However, these atrial waves are minute components in the ECG signal and can easily be distorted by noise, potentially undermining the algorithm’s efficacy.

Ventricular activity in AF is characterized by the inconsistency in RR intervals in the ECG signal, which relates to the timing difference of the QRS wave group [18, 39–44]. Algorithms based on this are less vulnerable to noise and evaluate the irregularities in the RR sequence. While these are robust even without atrial data, their detection accuracy has limitations. Algorithms that merge atrial and ventricular analyses offer both resilience and enhanced performance [45].

### Deep learning-based atrial fibrillation detection algorithms

Inspired by advancements in deep learning in areas like computer vision, speech recognition, and natural language processing [46], researchers have started leveraging it for AF detection. The shift has moved from conventional machine learning methods to sophisticated deep learning techniques. Predominantly, AF detection models using deep learning are built on Recurrent Neural Networks (RNN) [16, 25–27] and Convolutional Neural Networks (CNN) [16, 25, 47].

For CNN applications in AF detection, there are two main strategies. The first transforms the ECG signal into a time-frequency representation [14, 16, 25, 48]. The second employs a 1D-CNN [49] that directly interprets the one-dimensional ECG data.

While RNNs aren’t ideal for direct AF detection, their combination with CNNs, forming RCNNs, is gaining traction [14–16]. Typically, the ECG morphology segment utilizes a CNN or RCNN, processing the complete ECG signal to distill its cardiac features. Simultaneously, the rhythm component, often composed of one or more RNN layers, processes Heart Rate Variation (HRV) data to extract rhythm characteristics.

### Some wearable atrial fibrillation detection devices

In recent years, the functionality of smartwatches has extended beyond simple timekeeping to include health monitoring, particularly cardiac monitoring, making them a focal point in the realm of digital health. Representative smartwatches like the Apple Watch [50] incorporate built-in photoplethysmography sensors that can identify irregular pulses and detect atrial fibrillation and atrial flutter. These devices not only generate and record single-lead electrocardiograms (ECGs) when worn as recommended (on the left wrist), but also allow users to share the data with healthcare professionals through smartphone applications, thereby increasing the detection rate of cardiac arrhythmias and contributing to improved digital health outcomes.

A study sponsored by Apple in 2019 revealed that out of 450 participants, 34% received notifications of irregular pulses, leading to a diagnosis of atrial fibrillation in some cases [51]. While further testing is required to validate these findings, they support the effectiveness of smartwatches in detecting cardiac arrhythmias. Furthermore, research reports on the Apple Watch Series 4 and subsequent models suggest that these devices can be adjusted to record ECGs equivalent to the six positions of a traditional 12-lead ECG, including Einthoven’s leads I, II, III, and chest leads V1, V4, and V6, with comparable accuracy and signal quality [52, 53]. The application of this technology was further demonstrated in a case series study conducted in 2019, where the 3-lead ECGs reported by the Apple Watch consistently indicated ST-segment elevation, a marker for myocardial infarction, similar to conventional ECG readings [54], indicating the potential of these devices in early detection of acute coronary syndromes.

In addition to the Apple Watch, there are other wearable devices designed specifically for cardiac rhythm monitoring, such as AliveCor [55], ZioPatch [56], and ECG Check [57]. These devices transmit electrocardiogram data to smartphones via sensors for detecting atrial fibrillation, showcasing the integration of medical technology with mobile connectivity. Other innovative products, such as MyDiagnostick [58], simplify atrial fibrillation detection with its wand-like design, while the T-Shirt-Type Wearable Electrocardiography Monitor [59] embeds electrodes into fabric, providing continuous ECG monitoring for individuals leading active lifestyles. These developments demonstrate the progress of wearable ECG technology, combining medical accuracy with user convenience. They not only enhance the convenience and efficiency of cardiac health monitoring but also open up new possibilities for personal health management.

## Methodology

AF-MSDC (Atrial Fibrillation Detection using Multi-Scale Dilated Convolution) network is primarily composed of Dilated Convolution layers. As mentioned earlier, Dilated Convolution is a special type of convolutional layer that introduces a dilation factor *d*. Unlike traditional convolutional layers where the spacing between input elements matched by the convolutional kernel is 0, in Dilated Convolution, the spacing becomes *d* − 1 during each convolution operation. Traditional convolutional layers have a dilation factor of 0.

AF-MSDC network is an innovative network proposed as an alternative to the 1D-ResNet atrial fibrillation detection network. 1D-ResNet suffers from a large number of parameters due to its large kernel size and deep network structure. Simply reducing the kernel size would result in a decrease in the receptive field during convolution, leading to a decline in atrial fibrillation detection performance. However, reducing the kernel size is crucial for reducing the number of parameters in the convolutional network. To address this issue and maintain the receptive field size while reducing the kernel size, Multi-Scale Dilation Convolution is introduced to construct the AF-MSDC atrial fibrillation detection network.

1D-ResNet is primarily composed of 15 stacked Residual Blocks. In this study, various MSDC (Multi-Scale Dilated Convolution) blocks are designed to replace the Residual Blocks for constructing the AF-MSDC network. The figure below illustrates a structural comparison between the three types of MSDC blocks (MSDC block A, MSDC block B, and MSDC block C) and the Residual Block. In the figure, *k*, *s*, and *f* represent the kernel size, stride, and number of filters in the convolutional layer, respectively. For the MSDC block, the *k*, *s*, and *f* of the Dilated Convolution represent the kernel size, dilation factor, and number of filters, respectively. The stride *s* for Dilated Convolution is set to 1.

The three MSDC blocks correspond to the construction of AF-MSDC A, AF-MSDC B, and AF-MSDC C for atrial fibrillation (AF) detection networks. Table 1 provides a comparison between the three MSDC blocks and the Residual block, including the parameters of the convolutional layers, receptive field size, and network parameter sizes. The convolutional layer parameters (*k*, *s*, *d*, *f*) correspond to the kernel size, stride, dilation factor, and number of kernels, respectively. The receptive field calculation method is illustrated in Fig 2, where the number of sequence elements involved in the convolutional operation for each block is counted for a heart rate signal of length 16. From the figure, it can be observed that the receptive field sizes of the Residual block and the three MSDC blocks are 16, 13, 11, and 8, respectively.

Given the convolutional layer parameters (*k*, *s*, *d*, *f*), the formula to calculate the number of parameters *W* in the convolutional layer is as follows Formula (1).

$$\begin{array}{c} W=k*f \end{array}$$ (1)

Based on Formula (1), the number of parameters in the Residual block and the three MSDC blocks can be calculated as follows: 512 for the Residual block, 224 for MSDC block B, 128 for MSDC block A, and 256 for MSDC block C. From Table 1, it can be observed that MSDC block B has three dilated convolution branches, with a receptive field size closest to that of the Residual block, while having only one-fourth of the parameters compared to the Residual block. MSDC block A has two dilated convolution branches, with a receptive field size of 11/16 of the Residual block and less than half of the parameters. MSDC block C is a single-branch dilated convolution network, with both the receptive field size and the number of parameters being half of the Residual block. The AF-MSDC A, AF-MSDC B, and AF-MSDC C atrial fibrillation detection networks consist of 15 MSDC block A, MSDC block B, and MSDC block C, respectively. It can be inferred that the network parameters of AF-MSDC A and AF-MSDC B are half and one-fourth, respectively, compared to 1D-ResNet.

## Experiments

In this section, we first introduce two widely used datasets. Then, the experiment settings are described. The results of the datasets are reported and discussed at the end.

### Datasets and settings

This segment presents the evaluation results concerning the AF-MSDC algorithm on the MIT-BIH AFDB and the Physionet Challenge 2017 databases, sequentially. Utilizing the Adam optimization technique with an initial learning rate of 0.0001 for a duration of 100 cycles constitutes the training approach for AF-MSDC. Termination of training occurs promptly if the validation set’s loss does not decrease over a span of 10 successive epochs. Additionally, in the process of generating the cardiac waveform diagram, the adjacency matrix is assigned a value of 3. The comparison of relevant parameters between the MIT-BIH AFDB and the Physionet Challenge 2017 datasets is shown in Table 2.

**Table 2:** The comparison of relevant parameters between the MIT-BIH AFDB and the Physionet Challenge 2017 datasets.

	MIT-BIH AFDB	Physionet Challenge 2017
Segment Length(s)	10	9-30
Number of segments	82660	8528
Sampling rate	250HZ	300HZ
Signal bandwidth	0.1 ∼ 40HZ	-
Number of sessions	2	4
Dataset split ratio	Training set:8 Verification set:1 Test set:1	Training set:8 Verification set:1 Test set:1

MIT-BIH AFDB: Comprising 25 extended ambulatory ECG recordings from patients afflicted with AF, the MIT-BIH AFDB stands as a prominent public database in the domain of atrial fibrillation [60]. Each ECG recording spans approximately 10 hours and encompasses ECG data derived from leads II and V2. The sampling frequency employed is 250 Hz, while the resolution of ECG amplitude reaches 12 bits. Beth Israel Hospital in Boston served as the source for all ECG recordings, acquired using the Holter ambulatory ECG recorder, featuring a signal bandwidth spanning from 0.1 Hz to 40 Hz. The repository of MIT-BIH AFDB encompasses four principal rhythms, specifically the atrial fibrillation rhythm, atrial flutter rhythm, junctional rhythm, and normal rhythm. Among its contents are 65 labeled rhythm segments, inclusive of 12 junctional rhythm segments, 14 atrial flutter rhythm segments, and 291 atrial fibrillation rhythm segments, alongside an additional 288 segments presenting various other rhythms, including the normal rhythm.

In this study, we focused on leveraging the extensive MIT-BIH AFDB ECG dataset, which comprises extended recordings of electrocardiograms. Our approach involved segmenting these ECG recordings into 10-second intervals, forming the foundation of our AF dataset. This curated dataset was pivotal for evaluating the performance of our atrial fibrillation algorithm. It was imperative that the 10-second atrial fibrillation segments conformed entirely to the rhythmically labeled sections of the MIT-BIH AFDB. Similarly, the 10-second non-AF segments had to fit entirely within the annotated non-AF rhythm portions of the MIT-BIH AFDB. Through this systematic methodology, we successfully extracted a total of 82,660 signal segments, each lasting 10 seconds. Within this set, 33,072 segments corresponded to AF signals, while the remaining 49,588 segments represented non-AF signals. Notably, the AF and non-AF categories on the MIT-BIH AFDB aligned perfectly with the distribution of atrial fibrillation and non-AF segments. Subsequently, we partitioned these segments into training, validation, and testing sets using an 8:1:1 ratio.

The aim of categorization in the MIT-BIH AFDB dataset involves segregating ECG signals into two distinct groups: atrial fibrillation (AF) and non-atrial fibrillation (Non-AF). The corresponding metrics denoting index sensitivity and specificity are outlined in the subsequent manner:

$$\begin{array}{l} Sensitivity=N_{TP}/(N_{TP}+N_{FN})\\ Specificity=N_{TN}/(N_{TN}+N_{FP}) \end{array}$$ (2)

Where *N*_*TP*_ signifies the count of samples assigned as AF fragments within the set of AF fragment samples, *N*_*TN*_ corresponds to the tally of samples slated as non-AF fragments among non-AF segment samples, *N*_*FP*_ stands for the number of samples labeled as AF samples within the non-AF segment sample group, and *N*_*FN*_ denotes the figure of samples categorized as non-AF fragments.

Physionet Challenge 2017: The AFDB dataset from the Physionet Challenge 2017, known as the Physiological Data Challenge 2017 [61], constitutes a publicly accessible resource. Contributed by AliveCor, a company specializing in wearable ECG devices, the Cinc17 AFDB dataset encompasses ECG recordings gathered during the Physionet Challenge 2017. These ECG recordings, acquired at a sampling rate of 300 Hz, align more suitably with scenarios involving ECG wearable devices for AF monitoring. The ECG data within the Cinc17 AFDB repository are categorized into four classes: normal rhythm, atrial fibrillation rhythm, other rhythms, and noise recordings. These categories are labeled as Normal, AF, Other, and Noise respectively. The Physionet Challenge 2017 AFDB dataset is divided into two main segments: the training set and the test set. Inclusive of 8528 ECG records, the training set features ECG durations spanning from 9 to 30 seconds. Correspondingly, the test set, which mirrors the temporal distribution of the training set, comprises 3658 ECGs. As of now, the test set has not been publicly released.

The Physionet Challenge 2017 AFDB comprises ECG data characterized by short-range recordings, presenting sequences of non-segmented fixed-length signals. This dataset is organized into four distinct classification groups: normal cardiac rhythm, atrial fibrillation (AF) rhythm, alternate rhythms, and noise recordings. These categories are denoted as Normal, AF, Other, and Noise, respectively. The allocation of data involves partitioning into training, validation, and testing subsets, distributed at an 8:1:1 ratio.

The objective of the 2017 Physionet Challenge lies in the classification of ECG signals, a task centered at the intersection of artificial intelligence and bioinformatics. The primary goal is to assign ECG signals to one among four distinct categories: Normal, AF, Other, and Noise. This task entails a categorization complexity across four classes. In this investigation, the evaluation metrics encompass the macro-averaged F1 score alongside the F1 scores for individual categories denoted as *F*1_*all*_. Specifically, the F1 score corresponding to Normal rhythm is denoted as *F*1_*n*_, while the F1 score for AF rhythm is referred to as *F*1_*a*_. Furthermore, the F1 score related to other rhythms is recognized as *F*1_*o*_, whereas the F1 score pertaining to noise recordings is identified as *F*1_*p*_. Employing the F1 score offers a more equitable criterion for categorization, effectively balancing Sensitivity and Specificity and thereby mitigating the influence of class imbalance.

The F1 scores of the normal rhythm *F*1_*n*_,

$$\begin{array}{c} F1_{n}=\frac{2\times N_{n}}{\left(\Sigma_{N}+\Sigma_{n}\right)} \end{array}$$ (3)

The F1 scores of the AF rhythm *F*1_*a*_,

$$\begin{array}{c} F1_{a}=\frac{2\times A_{a}}{\left(\Sigma_{A}+\Sigma_{a}\right)} \end{array}$$ (4)

The F1 scores of the other rhythms *F*1_*o*_,

$$\begin{array}{c} F1_{o}=\frac{2\times O_{o}}{\left(\Sigma_{O}+\Sigma_{o}\right)} \end{array}$$ (5)

The F1 scores of the noise recordings *F*1_*p*_,

$$\begin{array}{c} F1_{p}=\frac{2\times P_{p}}{\left(\Sigma_{P}+\Sigma_{p}\right)} \end{array}$$ (6)

The final macro-average F1 score was calculated as,

$$\begin{array}{c} F1_{all}=\frac{1}{4}\left(F1_{n}+F1_{a}+F1_{o}+F1_{p}\right) \end{array}$$ (7)

### Results analysis and comparison

This section presents the evaluation results of the AF-MSDC algorithm on databases such as MIT-BIH AFDB and Physionet Challenge 2017, with a particular emphasis on highlighting the advantages of the AF-MSDC algorithm in terms of network parameter quantity.

The Atrial Fibrillation (AF) detection task on the MIT-BIH AFDB is a binary classification task, aiming to classify AF and non-AF signals, with evaluation metrics including sensitivity [62] and specificity [63]. Table 3 shows the evaluation results of the AF-MSDC algorithm on the MIT-BIH AFDB database, comparing it with other atrial fibrillation detection methods based on atrial activity [34], ventricular activity [39, 40, 42], combined atrial and ventricular activity [48], and deep learning methods [25–28, 43, 64–66].

**Table 3:** Comparison results of the AF-MSDC algorithm evaluated on MIT-BIH AFDB.

	Se(%)	Sp(%)	Method
J. Slocum et al. [34]	62.80	77.46	**Atrial Activity**
Dash et al. [40]	94.40	95.10	**Ventricular Activity**
Tateno et al. [39]	94.40	97.20	**Ventricular Activity**
Huang et al. [42]	96.10	98.10	**Ventricular Activity**
Jiang et al. [48]	98.20	97.50	**Atrial & Ventricular**
Xia et al. [25]	98.34	98.24	**2D CNN**
Xie et al. [26]	98.75	98.70	**Bi-direction LSTM**
Hannun et al. [27]	99.41	99.61	**1D-ResNet**
Zhou et al. [43]	96.89	98.25	**Recursive Algorithm**
Acharya et al. [28]	99.13	81.44	**CNNs**
Andersen et al. [64]	98.96	86.0	**CNNs-RNNs**
Ma et al. [65]	97.34	97.08	**CNN-LSTM**
Ma et.al. [66]	98.79	99.04	**iDCCN**
AF-MSDC A	**99.45**	**99.64**	**MSDC block A**
AF-MSDC B	99.45	99.61	**MSDC block B**
AF-MSDC C	99.15	99.38	**MSDC block C**

From the Table 3, it is evident that traditional methods, such as J. Slocum’s Atrial Activity [34], underperform other algorithms in both sensitivity and specificity. Notably, its sensitivity is only 62.80%, suggesting that this method might face challenges in detecting arrhythmias. Given that it primarily focuses on atrial activities, this may imply that atrial activities alone might not be sufficient to capture all instances of arrhythmia comprehensively. With the substantial advances made by deep learning-based methods in recent years, their performance on this task has significantly surpassed traditional approaches. For instance, the Ventricular Activity methods proposed by Dash et al. [40], Tateno et al [39], and Huang et al. [42] all achieve over 95% on Sp, demonstrating robust negative class prediction capabilities. The method by Jiang et al. [49], which integrates both atrial and ventricular activities, exhibits Se and Sp nearing 98%, suggesting that combining multiple cardiac activities could yield superior results. Some of the latest techniques in deep learning, like the 2D CNN by Xia et al. [25], 1D-ResNet by Hannun et al. [27], and Bi-directional LSTM by Xie et al. [26], have both Se and Sp exceeding 98%, showcasing their potential in detecting complex arrhythmic patterns. In terms of atrial fibrillation (AF) detection performance, our proposed two models based on multi-scale atrous convolutions, AF-MSDC A and AF-MSDC B, performed the best, surpassing the 1D-ResNet method. However, AF-MSDC C’s performance was similar to that of the state-of-the-art 1D-ResNet AF detection algorithm, with a slight decrease in performance. This could be attributed to AF-MSDC C only utilizing a single scale of dilated convolution blocks, leading to a reduced receptive field. This reduction introduces gaps in the input data, affecting the model’s effectiveness in feature extraction, potentially overlooking critical local features. The single dimension of atrous convolution also reduces the size of parameters, possibly failing to learn more complex representations, ultimately leading to decreased performance. However, our proposed multi-scale dilated convolution blocks effectively compensated for this loss in performance. Moreover, our models not only achieved industry-leading performance but also realized extreme reduction in the number of parameters. As shown in Table 4, the unit of network parameters is million (M). A comparison reveals that the number of parameters in AF-MSDC B is only a quarter of that in 1D-ResNet, yet its performance in AF detection is even better. The number of parameters in AF-MSDC A is about half of that in 1D-ResNet, yet it achieved the best AF detection performance. This fully demonstrates our success in maximizing performance with minimal resource consumption.

**Table 4:** Comparison of network parameter quantities between the three AF-MSDC atrial fibrillation detection networks and the 1D-ResNet.

	Se(%)	Sp(%)	Parameter size(M)
1D-ResNet [27]	99.41	99.61	121M
AF-MSDC A	99.45	99.64	54M
**AF-MSDC B**	**99.45**	**99.61**	**32M**
AF-MSDC C	99.15	99.38	61M

The ECG recordings in the MIT-BIH AFDB database are obtained from dynamic Holter ECG data and do not belong to data collected from wearable ECG devices. To validate the atrial fibrillation (AF) detection performance of the AF-MSDC algorithm on wearable ECG data, we evaluated the algorithm on the Physionet Challenge 2017 AFDB database. The ECG data in the Physionet Challenge 2017 AFDB are collected using the Kardia Band, a wearable ECG device developed by AliveCor. This dataset is suitable for assessing the algorithm’s performance on wearable ECG data. The AF detection on the Physionet Challenge 2017 AFDB involves a four-class classification task, aiming to classify the input ECG signals into Normal, AF, Other, and Noise categories. The evaluation metrics include the Normal F1 Score ($F_{1_{n}}$), AF F1 Score ($F_{1_{a}}$), Other F1 Score ($F_{1_{o}}$), Noisy F1 Score ($F_{1_{p}}$), and the average F1 Score ($F_{1_{all}}$). Table 5 presents the evaluation results of the AF-MSDC algorithm on the Physionet Challenge 2017 AFDB database. A comparison is made with existing AF detection algorithms developed using real wearable ECG data, including deep learning-based algorithms [14, 15, 27, 67] and machine learning-based algorithms [16, 19].

**Table 5:** The comparative evaluation results between the AF-MSDC algorithm and existing atrial fibrillation (AF) detection algorithms on the Physionet Challenge 2017 AFDB dataset.

	*F*1_*n*_	*F*1_*a*_	*F*1_*o*_	*F*1_*p*_	*F*1_*all*_	Method
Radovan [16]	90.56%	85.47%	75.82%	73.39%	81.31%	SVM
Yazdan [19]	94.27%	81.75%	84.19%	60.88%	80.27%	SVM
Xiong [14]	92.64%	87.74%	82.71%	68.81%	82.98%	2D-GoogLeNet
Hannum [27]	99.70%	90.10%	82.10%	62.50%	83.60%	1D-ResNet
Philip [15]	92.84%	83.96%	81.50%	58.82%	79.28%	1D-RCNN
Limam [67]	92.92%	83.47%	81.37%	70.18%	81.99%	RCNN+HRV
**AF-MSDC A**	**99.70%**	**88.60%**	76.40%	**77.80%**	**85.63%**	**MSDC block A**
AF-MSDC B	99.50%	85.90%	76.10%	77.80%	84.83%	MSDC block B
AF-MSDC C	99.60%	87.10%	77.60%	70.60%	83.73%	MSDC block C

From Table 5, it is evident that compared to atrial fibrillation detection algorithms based on deep learning, atrial fibrillation detection algorithms based on machine learning, such as SVM methods by Radovan and Yazdan, perform relatively weaker. Specifically, on the *F*1_*all*_ evaluation metric, the highest score achieved by SVM methods is only 81.31%, whereas scores of deep learning methods significantly exceed this value. Among the deep learning algorithms, 1D-RCNN, when combined with HRV (1D-RCNN+HRV), achieves an *F*1_*all*_ score of 81.99%, which is 2.71 percentage points higher than 1D-RCNN score without HRV (79.28%). This clearly underscores the crucial role of heart rate variability (HRV) as an additional cardiac rhythm feature in enhancing AF detection performance.

Among the listed deep learning-based AF detection algorithms, 1D ResNet boasts the most remarkable *F*1_*all*_ performance, reaching 83.60%. However, in comparison, the performance of proposed algorithms AF-MSDC A and AF-MSDC B stands out even more. Specifically,AF-MSDC A achieves an *F*1_*all*_ score of 85.63% (as shown in Table 6) with less than half the number of parameters compared to 1D-ResNet, which is significantly higher than that of 1D ResNet. Additionally, AF-MSDC B still outperforms 1D-ResNet even with only a quarter of the parameters of 1D-ResNet, reaching an *F*1_*all*_ score of 84.83%. This superior performance can be attributed to the design of the MSDC block within AF-MSDC. Through this design, the network can extract multi-scale features from ECG signals, leading to stronger and more discriminative classification features, which further enhances the performance of AF detection.

**Table 6:** Comparison of network parameter quantities between the three AF-MSDC atrial fibrillation detection networks and the 1D-ResNet.

	*F*1_*all*_	Parameter size(M)
1D-ResNet [27]	83.60%	121M
AF-MSDC A	85.63%	54M
**AF-MSDC B**	**84.83%**	**32M**

When comparing the performance of two multi-scale convolutional models, AF-MSDC A and AF-MSDC B, on two datasets (Tables 3 and 5), we observed that AF-MSDC A, which has fewer types of dilated convolutional kernels(Table 1), actually outperformed AF-MSDC B (both models have an equal number of dilated convolutional kernels, denoted as F, corresponding to the number of convolutional kernels in 1D-ResNet). We hypothesize that although AF-MSDC B has a sparser structure, more expansive convolutions, and a larger receptive field(Table 1), theoretically enabling it to capture a wider range of features from electrocardiogram (ECG) signals, these features may not be as significantly effective for AF detection as the richer and more important scale features captured by AF-MSDC A. This highlights the importance of striking a balance between the quantity of multi-scale features and the fundamental scale features that are crucial for the task.

## Conclusion

Atrial Fibrillation (AF) is clinically pivotal due to its association with increased risks, notably those pertaining to age-induced stroke events. Addressing this crucial concern, our research elucidates a novel approach harnessing a multi-dilation Convolutional Neural Network (CNN) enhanced with Multi-Scale Dilation Convolution (MSDC) blocks. This innovative methodology sets itself apart by its ability to adeptly extract multi-scale features, thus achieving marked parameter optimization. The effectiveness of our methodology receives affirmation through meticulous assessments employing well-recognized databases, such as the MIT-BIH Atrial Fibrillation Database and the Physionet Challenge 2017. Impressively, in comparison to the ResNet model, our approach consistently demonstrates superior performance while maintaining a significantly reduced parameter size.

Meanwhile, our model exhibits enormous application potential, capable of being integrated into wearable atrial fibrillation detection devices while minimizing hardware requirements, thereby reducing the risk of data breaches and ensuring the best possible detection outcomes.
